# Primary Hyperoxaluria Type 1 Diagnosed After Kidney Transplantation in the Absence of Classical Features

**DOI:** 10.1155/crin/4378614

**Published:** 2026-08-29

**Authors:** Rawh Sultan, Zein A. Alsayed-Ahmad, Ahmad Ryyan Shheibar, Rawda Shawwa, Karam Albitar, Sami Albitar

**Affiliations:** ^1^ Faculty of Medicine, Aleppo University, Aleppo, Syria, alepuniv.edu.sy; ^2^ Faculty of Medicine, Syrian Private University, Damascus, Syria, spu.edu.sy

**Keywords:** adult-onset, AGXT mutation, genetic testing, kidney transplantation, oxalate nephropathy, primary hyperoxaluria Type 1

## Abstract

**Background:**

Primary hyperoxaluria Type 1 is a rare inherited metabolic disorder caused by pathogenic variants in the AGXT gene. Because the disorder leads to excessive internal oxalate formation, progressive end‐stage kidney disease (ESKD) may occur. While most affected individuals present during childhood, a subset is diagnosed only later in adult life, frequently after substantial renal deterioration. Delayed diagnosis increases the risk of recurrent oxalate nephropathy and graft dysfunction following kidney transplantation.

**Case Presentation:**

We report a 30‐year‐old woman with ESKD of unknown aetiology who underwent living‐donor kidney transplantation. One year later, during a subsequent pregnancy, she developed progressive graft dysfunction. Kidney biopsy demonstrated extensive calcium oxalate crystal deposition, raising suspicion for an underlying metabolic disorder. Genetic testing subsequently confirmed a pathogenic AGXT mutation, establishing the diagnosis of adult‐onset PH1. Urinary oxalate concentrations were within normal limits before and after transplantation, emphasizing the diagnostic challenge.

**Conclusion:**

This case highlights the importance of considering PH1 in adults with unexplained chronic or ESKD, even in the absence of classical features or elevated urinary oxalate excretion. Early genetic testing and careful assessment of family history are essential for timely diagnosis, enabling appropriate transplant planning, disease‐specific therapy, and genetic counselling.

## 1. Introduction

Systemic oxalosis represents a late‐stage complication of primary hyperoxaluria (PH), a rare autosomal recessive disorder caused by pathogenic variants affecting glyoxylate metabolism. These defects result in excessive oxalate production, leading to calcium oxalate crystal deposition, mainly within renal tissues [1, 2].

PH is categorized into three distinct types: PH1, PH2, and PH3 based on the affected enzyme involved [1, 3]. PH1 is the most severe and common subtype, presenting during childhood with recurrent nephrolithiasis, nephrocalcinosis, or progressive kidney dysfunction [3]. However, nonspecific symptoms and the absence of a history of kidney stones may delay diagnosis, particularly in adult patients.

In undiagnosed cases, oxalosis may become evident shortly after kidney‐only transplantation, as stored oxalate is mobilized and deposited within the new graft. However, in this patient, there was stable graft function for more than 1 year before oxalosis developed during a subsequent pregnancy. This case highlights the diagnostic challenge of late‐onset PH1 and the importance of early metabolic and genetic evaluation in patients with end‐stage kidney disease (ESKD) of uncertain origin, particularly before transplantation.

## 2. Case Presentation

A 30‐year‐old primigravida presented during the fourth month of pregnancy with worsening fatigue. Obstetric ultrasound confirmed intrauterine fetal demise, estimated to have occurred approximately 1 month earlier. After medical termination, laboratory and ultrasound revealed underlying chronic kidney disease (CKD), with a serum creatinine of 10 mg/dL and bilaterally small kidneys.

The patient was started on regular hemodialysis and remained dialysis‐dependent for 6 months until she underwent a living‐donor kidney transplant from a 2/6 HLA‐matched relative. Maintenance immunosuppressive therapy consisted of tacrolimus (dose adjusted to maintain trough levels), mycophenolate mofetil 500 mg twice daily, and prednisolone 7.5 mg daily. Posttransplant recovery was uneventful, and graft function remained robust with a baseline serum creatinine of 1.1–1.3 mg/dL during the first year.

Approximately 1 year after transplantation, the patient became pregnant again. Because mycophenolate mofetil is contraindicated during pregnancy, it was discontinued and replaced with azathioprine. The pregnancy progressed normally until the late second trimester, when she began to develop signs of worsening renal function, including elevated blood pressure, mild lower limb edema, and a moderate rise in serum creatinine (peaking at 4.32 mg/dL). Intravenous methylprednisolone 500 mg daily was added in response to the suspected graft dysfunction. After delivery, serum creatinine levels remained mildly elevated, but there was no evidence of complete graft failure. A follow‐up renal ultrasound demonstrated an enlarged transplanted kidney with edema of the pyramids and thickening of the collecting system walls—findings that, while nonspecific, can be associated with graft rejection. However, color Doppler and pulse wave assessments remained within normal limits.

**FIGURE 1:**
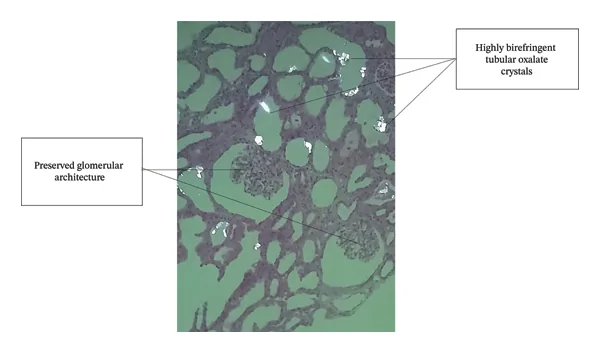
Low‐power polarized light microscopy of the renal allograft biopsy demonstrating preserved glomerular architecture with patent capillary loops and no significant glomerular abnormalities. The surrounding cortical tubules are dilated with mild interstitial inflammatory infiltrate, consistent with acute tubular injury. Highly birefringent calcium oxalate crystals are diffusely scattered within the tubular lumens.

**FIGURE 2:**
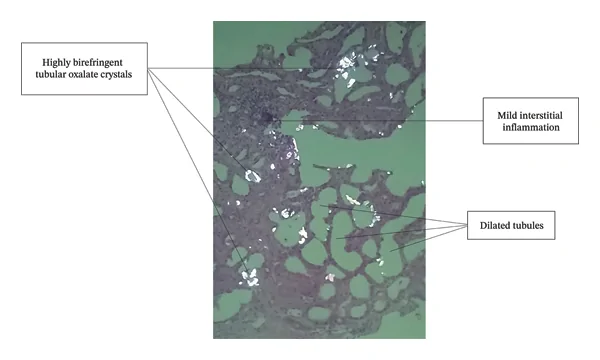
Polarized light microscopy of the renal allograft biopsy showing a well‐preserved glomerulus with patent capillary loops and unremarkable mesangial areas under polarized light. No active inflammation, glomerular sclerosis, or interstitial fibrosis is identified. Intratubular birefringent calcium oxalate deposits are visible in the surrounding parenchyma.

The biopsy of the renal allograft showed acute tubular necrosis with tubulointerstitial inflammation and widespread deposition of crystals. Polarized light microscopy identified numerous birefringent calcium oxalate crystals within tubular lumina and interstitium (Figures 1 and 2), raising suspicions for a metabolic disorder.

**FIGURE 3:**
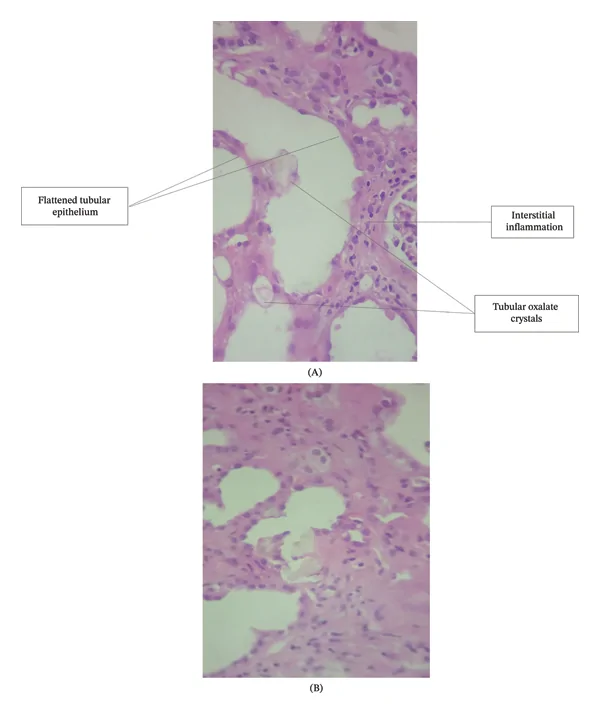
High‐power view of an H&E‐stained section of the renal allograft biopsy demonstrating intratubular deposition of refractile calcium oxalate crystals associated with acute tubular injury represented by flattened epithelium; there is also mild interstitial inflammatory infiltrate and edema.

High‐power evaluation confirmed acute tubular injury with intratubular crystal aggregates and associated epithelial degeneration (Figure 3).

Urinary oxalate levels measured both before and after transplantation were within normal or undetectable ranges, likely due to the kidney’s limited excretory function and systemic oxalate accumulation. Plasma oxalate measurement was not available at the time of evaluation. Genetic testing was performed because of the unusual clinical presentation and suggestive family history, including a sister with recurrent pregnancy loss and elevated creatinine levels and also a maternal aunt requiring chronic dialysis.

Genetic testing identified a homozygous AGXT c.33_34insC (p.Lys12fs) frameshift variant in exon 1, confirming the diagnosis of PH Type 1 (PH1). Because this truncating variant is not associated with pyridoxine responsiveness, a clinically meaningful response to vitamin B6 was considered unlikely. Although the biopsy findings suggested an underlying metabolic disorder, the definitive diagnosis was established only through genetic analysis of AGXT.

Following the diagnosis, the patient was immediately started on an intensified hemodialysis regimen to maximize plasma oxalate clearance and was evaluated for combined liver–kidney transplantation. Despite intensive dialysis, allograft function continued to deteriorate, primarily because of ongoing hepatic oxalate production. Persistent hepatic oxalate production ultimately led to irreversible graft failure. No clinical evidence of cardiac oxalosis or retinal oxalate deposition was documented.

The patient subsequently required long‐term hemodialysis through a central venous catheter after developing central venous thrombosis. Despite close clinical surveillance, she developed a catheter‐related bloodstream infection that rapidly progressed to septic shock and multiorgan failure. This acute septic event was the immediate cause of death, occurring before advanced systemic manifestations of oxalosis became clinically apparent.

## 3. Discussion

Mutations in the AGXT gene, which encodes the hepatic enzyme alanine‐glyoxylate aminotransferase (AGT), cause PH1, an autosomal recessive metabolic disorder [4]. In healthy individuals, AGT converts glyoxylate to glycine, preventing oxalate accumulation. In patients with PH1, AGT deficiency diverts glyoxylate metabolism toward lactate dehydrogenase‐ and glycolate oxidase‐mediated pathways, resulting in oxalate overproduction [5].

PH1 can present at any age. Approximately 10% of patients are diagnosed in infancy, 70% during childhood or adolescence, and 20% in adulthood [6]. Although early symptoms often prompt metabolic evaluation, some patients, particularly adults, may remain asymptomatic until advanced renal failure [7]. Our case exemplifies this diagnostic challenge. The patient presented with advanced renal failure, undetectable urinary oxalate, and no history of stone disease. This atypical presentation contributed to a significant delay in diagnosis, which was established 1 year after kidney transplantation following graft dysfunction detected during pregnancy. This delay likely allowed oxalate accumulation within the allograft, resulting in irreversible damage and early graft dysfunction due to systemic oxalate burden [2]. This case underscores the importance of considering PH1 in patients with unexplained renal failure, even in the absence of classic clinical or biochemical features.

The patient’s family history played a critical role in establishing the diagnosis. Her aunt’s history of prolonged dialysis, along with her sister’s recurrent pregnancy loss with impaired renal function, suggested an inherited disorder. Genetic testing confirmed an AGXT mutation, establishing PH1 and highlighting the marked intrafamilial phenotypic variability of the disease despite a presumed shared pathogenic variant. This underscores the value of genetic testing for confirming the diagnosis, identifying affected relatives, guiding reproductive counselling, and informing transplant planning [8].

This case highlights a missed opportunity for disease‐specific treatment and appropriate transplant planning. Pyridoxine is most effective in selected patients with recognized responsive AGXT missense variants; treatment response should ideally be established by a sustained reduction in urinary oxalate excretion when kidney function permits assessment [6]. The patient’s homozygous c.33_34insC (p.Lys12fs) frameshift variant is predicted to result in a truncated protein and is not among the established pyridoxine‐responsive variants, making a clinically meaningful response unlikely, although direct functional data for this specific variant and a documented therapeutic trial were unavailable. Published transplant experience indicates that lumasiran, combined with intensive or prolonged dialysis, high fluid intake when feasible, and crystallization inhibitors can markedly reduce plasma oxalate concentration and may preserve renal allografts when PH1 is diagnosed after transplantation [2]. These reports support prompt initiation of RNA‐interference therapy when available; however, evidence in the posttransplant setting remains limited, and treatment should be individualized. For patients with adequately controlled oxalate production—historically those with confirmed pyridoxine responsiveness, and increasingly selected patients receiving effective RNA‐interference therapy—kidney‐alone transplantation may be considered in expert centers. In patients whose hepatic oxalate production is not adequately controlled, combined liver–kidney transplantation remains an established strategy because liver replacement corrects the AGT defect [6]. While liver transplantation remains the only definitive curative option for patients with PH1, RNA‐interference therapies, particularly subcutaneous lumasiran, now offer a potent means to reducing hepatic oxalate production in patients of all ages and stages of renal failure [9].

Prior to kidney transplantation, PH1 should be suspected in candidates with unexplained ESKD, particularly those younger than 40 years or with a history of nephrolithiasis, nephrocalcinosis, a positive family history, or oxalate crystal deposition on kidney biopsy. Importantly, the absence of kidney stones does not exclude PH1. In patients with advanced CKD or receiving dialysis, the diagnosis relies primarily on plasma oxalate measurement and genetic testing rather than urinary oxalate excretion [4, 6].

This case demonstrates that PH1 may remain unrecognized until recurrent oxalate nephropathy develops in a renal allograft. Normal or undetectable urinary oxalate in advanced kidney failure does not exclude PH1. In transplant candidates with unexplained ESKD, particularly at a young age or with a suggestive family history, pretransplant plasma oxalate measurement and genetic testing should be considered. Early diagnosis enables genotype‐informed therapeutic planning, including pyridoxine assessment, timely RNA‐interference therapy, and appropriate selection of kidney‐alone versus combined liver–kidney transplantation.

## 4. Conclusion

This case emphasizes the importance of considering adult‐onset PH1 in unexplained CKD. Genetic testing of the AGXT gene was essential for definitive diagnosis, guiding management and family counseling. Delayed recognition led to systemic oxalosis and graft dysfunction, highlighting the need for early metabolic and genetic evaluation to optimize outcomes.

**Nomenclature**
PH: Primary hyperoxaluria
PH1: Primary hyperoxaluria Type 1
PH2: Primary hyperoxaluria Type 2
PH3: Primary hyperoxaluria Type 3
AGT: Alanine‐glyoxylate aminotransferase
AGXT: Alanine‐glyoxylate aminotransferase gene
ESRD: End‐stage renal disease
HLA: Human leukocyte antigen
mg/dL: Milligrams per deciliter
mL/min: Milliliters per minute

## Author Contributions

Rawh Sultan, Ahmad Ryyan Shheibar, Rawda Shawwa, and Zein A. Alsayed‐Ahmad wrote the manuscript.

Zein A. Alsayed‐Ahmad critically revised and edited the manuscript.

Karam Albitar collected the data.

Sami Albitar diagnosed and treated the case.

## Funding

No funding was received for this manuscript.

## Disclosure

I would like to declare that the authors have prepared this submission in their personal capacity and not as an official representative or otherwise on behalf of a sanctioned government. All authors have reviewed and approved the final manuscript.

## Consent

Written informed consent was obtained from the patient’s next of kin for the publication of this case report and any accompanying images.

## Conflicts of Interest

The authors declare no conflicts of interest.

## Data Availability

The data that support the findings of this study are available on request from the corresponding authors. The data are not publicly available due to privacy or ethical restrictions.
